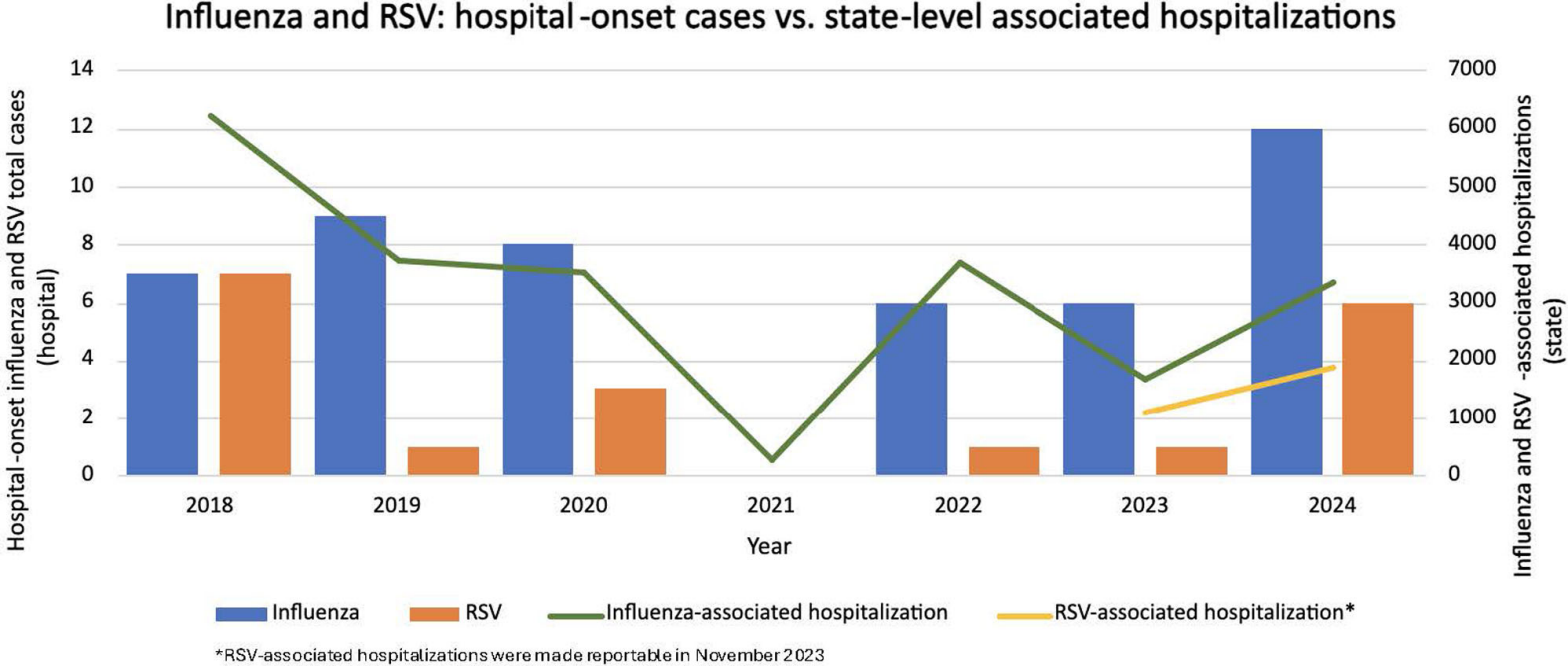# 370 Impact of Integrated Pharmacy/Infectious Diseases Physician Rounding on the Internal Medicine Teaching Service Antibiotic Stewardship

**DOI:** 10.1017/ash.2026.10706

**Published:** 2026-06-23

**Authors:** Emily Schmitz, Laura Anderson, Dan Shirley

**Affiliations:** 1 University of Wisconsin School of Medicine and Public Health; 2 UW Health

## Abstract

**Title:** Hospital-onset influenza and RSV: Impact of Infection Prevention Efforts During the COVID-19 Pandemic Authors: Emily Schmitz, MD, MPHa Laura Anderson, RN, MPH, CICb Daniel Shirley, MD, MSa,b,c Affiliations: University of Wisconsin School of Medicine and Public Health, Madison, WI UW Health, Madison, WI Division of Infectious Disease, Department of Medicine, University of Wisconsin School of Medicine and Public Health, Madison, WI Objective: To quantify and characterize hospital-onset influenza and respiratory syncytial virus (RSV) cases, pre- and post- COVID-19 pandemic. Design: Retrospective cohort study from January 1, 2018 - December 31, 2024. Setting: This study was conducted at a large urban Midwestern tertiary-care hospital system. Participants and Interventions: The Infection Prevention team reviewed the electronic medical records (EMR) of patients who were admitted > 48 hours and subsequently tested positive for influenza and/or respiratory syncytial virus (RSV). Incubation periods for each virus were determined based on Centers for Disease Control and Prevention (CDC) guidance and literature review. Hospital-onset was defined as a positive test three or more days after admission for influenza and six or more days after admission for RSV. Patients were excluded if a positive test did not meet these criteria or was completed at least 1-week prior to admission. Clinical data including medical comorbidities, anti-viral administration, vaccination and immunocompromised status were collected from the EMR. Hospital-associated influenza and RSV data were taken from the Wisconsin Communicable Disease Surveillance Data. Results Forty-nine patients met criteria for hospital-onset influenza infection, and 44 (89%) were symptomatic. Twenty-one (43%) patients required the intensive care unit (ICU) and mechanical ventilation during their hospitalization. The majority of patients (92%) received anti-viral therapy (oseltamivir). Nineteen patients (39%) were immunocompromised. A total of 30 (61%) were vaccinated against influenza for the season. Three patients died during their hospitalization, for an in-hospital mortality rate of 0.06%. Nineteen patients met criteria for hospital-onset RSV infection and 18 (95%) were symptomatic. In patients with RSV, 6 (32%) required the ICU and mechanical ventilation during their hospitalization. Only 5 patients (26%) received antiviral therapy for their RSV infection. Compared to influenza, hospital-onset RSV was associated with a higher in-hospital mortality rate (n=3, 16%). There were no hospital-onset influenza or RSV cases from April 2020 through March 2022, which coincides with the COVID-19 pandemic. Cases increased with 12 cases of hospital-onset influenza and 6 cases of hospital-onset RSV in 2024, which were similar to pre-pandemic rates. Discussion Hospital-onset influenza and RSV lead to significant morbidity and mortality. These preventable infections also put healthcare workers and other patients at risk. Data suggest that enhanced infection prevention efforts during the COVID-19 pandemic mitigated the number of hospital-onset cases of influenza and RSV. During periods when influenza and RSV rates are high, enhanced infection prevention efforts are likely warranted to prevent hospital-onset cases.

**Figure:**